# Effects of Acute or Chronic Ethanol Exposure during Adolescence on Behavioral Inhibition and Efficiency in a Modified Water Maze Task

**DOI:** 10.1371/journal.pone.0077768

**Published:** 2013-10-17

**Authors:** Shawn K. Acheson, Craig Bearison, M. Louise Risher, Sabri H. Abdelwahab, Wilkie A. Wilson, H. Scott Swartzwelder

**Affiliations:** 1 Neurobiology Research Laboratory, Durham Veterans Affairs Medical Center, Durham, North Carolina, United States of America; 2 Department of Psychiatry and Behavioral Sciences, Duke University Medical Center, Durham, North Carolina, United States of America; 3 Departments of Psychology and Neuroscience, Duke University, Durham, North Carolina, United States of America; 4 Social Sciences Research Institute, Duke University, Durham, North Carolina, United States of America; Université Pierre et Marie Curie, France

## Abstract

Ethanol is well known to adversely affect frontal executive functioning, which continues to develop throughout adolescence and into young adulthood. This is also a developmental window in which ethanol is misused by a significant number of adolescents. We examined the effects of acute and chronic ethanol exposure during adolescence on behavioral inhibition and efficiency using a modified water maze task. During acquisition, rats were trained to find a stable visible platform onto which they could escape. During the test phase, the stable platform was converted to a visible floating platform (providing no escape) and a new hidden platform was added in the opposite quadrant. The hidden platform was the only means of escape during the test phase. In experiment 1, adolescent animals received ethanol (1.0g/kg) 30min before each session during the test phase. In experiment 2, adolescent animals received chronic intermittent ethanol (5.0g/kg) for 16 days (PND30 To PND46) prior to any training in the maze. At PND72, training was initiated in the same modified water maze task. Results from experiment 1 indicated that acute ethanol promoted behavioral disinhibition and inefficiency. Experiment 2 showed that chronic intermittent ethanol during adolescence appeared to have no lasting effect on behavioral disinhibition or new spatial learning during adulthood. However, chronic ethanol did promote behavioral inefficiency. In summary, results indicate that ethanol-induced promotion of perseverative behavior may contribute to the many adverse behavioral sequelae of alcohol intoxication in adolescents and young adults. Moreover, the long-term effect of adolescent chronic ethanol exposure on behavioral efficiency is similar to that observed after chronic exposure in humans.

## Introduction

It is well established that ethanol has a detrimental effect on executive cognitive processes. For example, ethanol has been shown to increase perseverative errors in humans in both laboratory [1,2] and naturalistic settings [3], and to reduce response inhibition, though this effect can be altered by motivational factors [4]. Similar findings have been observed in animal models. For example, adult rats demonstrated marked perseveration in an 8-arm radial arm maze task following administration of 1.5 or 2.0g/kg ethanol, but not following vehicle or ethanol doses of 1.0g/kg [5] or 0.75g/kg [6]. Surprisingly, the long-term effects of chronic ethanol exposure on executive functions are less well understood. In general, the most severe executive function deficits observed after chronic ethanol abuse occur in individuals with alcohol induced Korsakoff’s syndrome. The prefrontal executive deficits observed in non-Korsakoff alcohol abuse patients are milder in nature and appear to be driven as much by age and duration of abuse and abstinence as they are by the severity of the abuse. The relative few studies in animal models indicate that chronic ethanol produces deficits in perseveration and cognitive flexibility [7,8], though it is challenging to establish behavioral tasks for rodents that model the higher order cognitive domains of executive function in humans.

 Executive functions including response inhibition, set shifting and perseveration are evolutionarily conserved behaviors largely attributed to the frontal lobes and their functional connectivity to other cortical regions [9]. Importantly, both executive functions and the cortical regions to which they are attributed continue to develop well into adolescence and beyond [10-13]. It is also important to note that these human developmental changes frequently coincide with the initial use of drugs of abuse [14]. 

There is now sound evidence that exposure to drugs of abuse (including ethanol) during adolescence can produce adverse and/or distinctive effects on cognition, behavior, and corresponding brain physiology. For example, adolescent animals appear less sensitive to the anxiogenic [15-17], locomotor impairing [18], sedative [19,20], and hypnotic [21] effects of acute ethanol. In addition, prior work from our laboratory has demonstrated that moderate ethanol doses (1.0 - 2.0 g/kg) may more potently inhibit hippocampally dependent learning in adolescent rats than their adult counterparts [22]. These effects were not observed with lower (0.5g/kg) or higher (2.5g/kg) doses of ethanol [23]. These findings are generally consistent with electrophysiological evidence demonstrating that ethanol more potently suppressed the induction of LTP and NMDA receptor-mediated synaptic activity, and enhanced extrasynaptic GABA_A_ receptor function in adolescent than adult hippocampal slices [24-27]. These findings are also generally consistent with human literature in which college aged humans (post-adolescent) were more susceptible to the memory impairing effect of ethanol than were middle-aged adults [28]. Despite these findings, the adolescent sensitivity to the memory impairing effects of ethanol have not been observed consistently across cognitive end points in the Morris water maze [29,30]; in alternate measures of spatial learning [31]; or across species [32].

The primary purpose of the present experiments was to better understand the neurodevelopmental effect of ethanol on perseveration and behavioral inhibition in adolescent rats. In a related study, mice chronically treated with ethanol during adolescence showed no impairment of initial acquisition on a spatial learning task or in acquisition of a new spatial referent following reversal [33]. However, there was evidence of performance deficits in spatial recall in a probe trial following reversal. Mice treated with chronic ethanol during adolescence spent less time searching the reversal quadrant than they did the initial spatial quadrant. Although difficult to interpret in the absence of information concerning the temporal sequence of the search strategy, it does suggest that those animals may have perseverated on the previous platform location in the absence of a tangible platform. 

Based on those findings we have developed a modified water maze task to maximize the establishment of a pre-potent response during the initial acquisition phase, which the animal is then required to inhibit during the test phase. Using this new methodology, we hypothesized that acute ethanol would promote perseverative behavior independent of hippocampally dependent spatial learning in adolescent animals (experiment 1). We further hypothesized, that animals exposed to chronic intermittent ethanol during adolescence would demonstrate similar perseverative deficits as adults (experiment 2). 

## Methods

### Animals

All procedures were reviewed and approved by the Duke University and Durham Veteran’s Administration Medical Center Institutional Animal Care and Use Committees. Male Sprague-Dawley rats (Charles River, Raleigh, NC) were housed on a reverse 12:12-hour light-dark cycle and were provided ad libitum access to food and water (experiment 1: n=16, experiment 2: n=24). In experiment 1, animals were handled daily for three consecutive days before behavioral procedures commenced at post-natal day 30 (PND30). In experiment 2, animals were handled twice daily for three consecutive days before dosing commenced on PND30. Animals were acclimated to the water maze by allowing free swim for 30s (experiment 1) or 60s (experiment 2) the day before the initial training. All behavioral testing was performed during the animals’ dark cycle. 

Post-natal day 30 was chosen as a model of adolescence based on an extensive literature demonstrating that rats undergo a developmental phase that is both physiologically [34] and behaviorally ([35,36]) similar to adolescence in humans. For example, the window from PND28 – PND50 in male rats represents a period of sexual maturation marked by the development and presence of mature sperm [34]. Similarly, Spear and colleagues have characterized a set of neurobehavioral criteria between PND28 and PND50 that are consistent with human adolescence [35]. Based on these and related findings, the age range between postnatal days 28 and 50 has most often been chosen to represent the adolescent period in the rat and animals beyond 70 days of ages are considered fully adult [35].

### Drug Preparation and Dosing

#### Experiment 1

Animals (n=8/group) were randomly assigned to receive either ethanol or isovolumetric saline (7.9mL/kg). USP grade laboratory ethanol (1g/kg, 16% v/v in normal saline) or sterile saline were administered via i.p. injection, 30 minutes before behavioral assessment in the test phase (PND34 - 41).

#### Experiment 2

Animals (n=12/group) were randomly assigned to receive either chronic intermittent ethanol (CIE) or isovolumetric saline (CIS). USP grade laboratory ethanol (5g/kg, 35% v/v in normal saline) or sterile saline were administered by gavage. Animals received a total of 10 CIE or CIS treatments administered over 16 days. Dosing occurred on Monday and Tuesday, and Thursday and Friday each week. Animals were then allowed to washout until they reached PND71. Behavioral testing was initiated on PND72. 

### Modified Water Maze

The purpose of this task was to assess the animal’s ability to inhibit a prepotent behavioral response using the Morris water maze apparatus. This task is conceptually similar to a reversal-learning task but was modified to maximize the animals’ tendency to perseverate on the initial platform location. In this version of the task, animals underwent initial training on a visible platform. The tank used in this experiment was 1.5m in diameter and filled with 22°C water to a depth of 0.5m. The rigid and floating platforms, identical in appearance, were 12cm in diameter and constructed of PVC. The rigid platform was affixed to a stand resting on the floor of the tank. The floating platform was tethered to a counter weight on the bottom of the tank so that it remained in the same location in each trial. The floating platform was also neutrally buoyant so that it would sink whenever an animal tried to climb aboard. The tether and buoyancy of the floating platform were constructed so that the platform would tilt toward the approaching animal and immediately begin to sink as the animal attempted to climb on top. 

During the acquisition phase, animals were trained to locate a rigid platform marked by a flag extending 30cm above the surface of the water. Acquisition involved two training sessions per day (three trials per session) for four consecutive days. Each trial was terminated at 60s or when the animal found the platform. Animals were gently guided to the visible platform on trials when they did not find it on their own. Animals were allowed to rest on the visible platform for 15 seconds before being removed to a warm dry towel for 30s between trials. 

**Figure 1:**
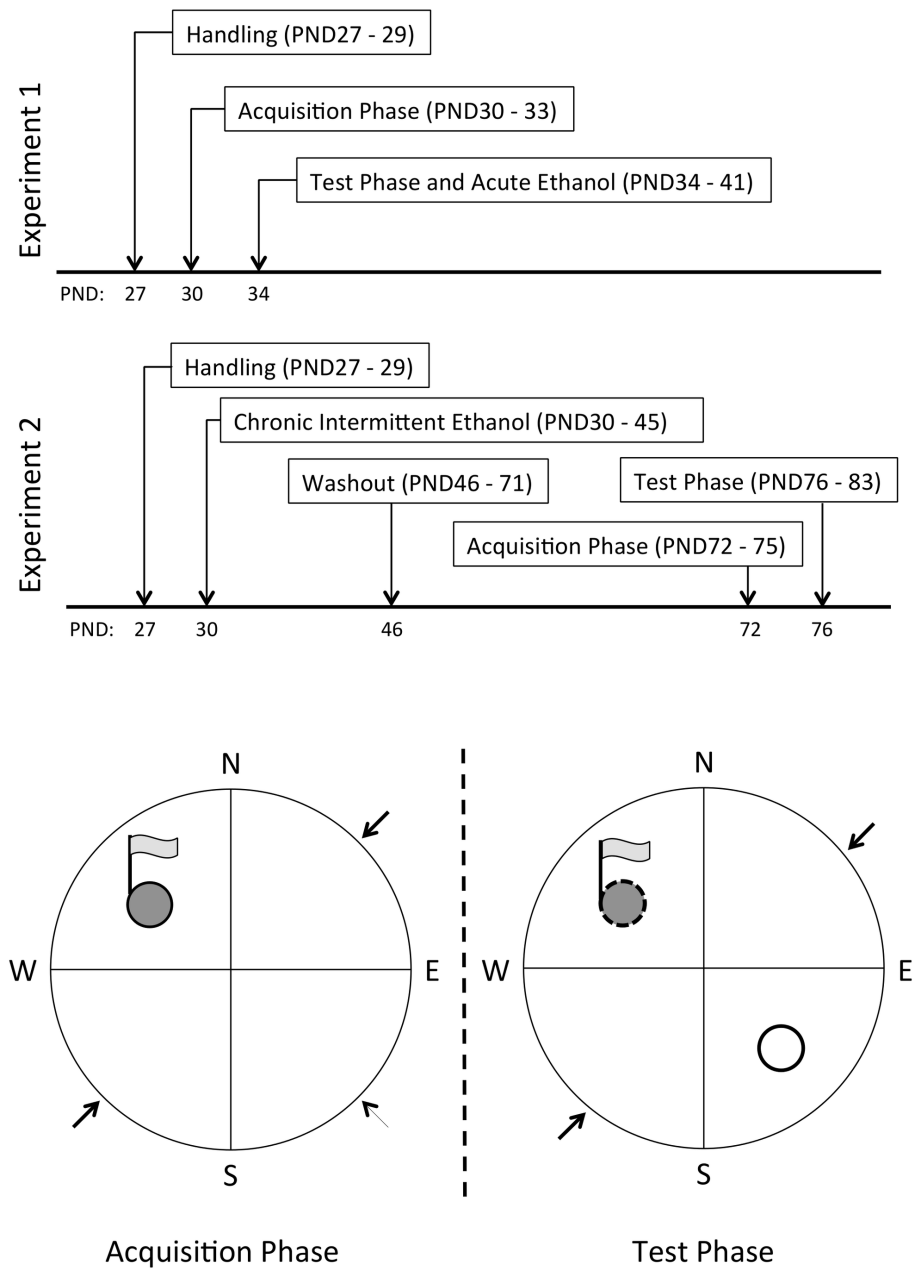
Timeline of events and synoptic diagram (experiments 1 and 2). Panel A depicts the postnatal day (PND) on which events and activities occurred. Panel B provides a schematic diagram of experimental apparatus during initial acquisition (IAP) and test phases (TP). During each phase, animals began each trial from one of the intermediate locations marked by the arrows. IAP: animals were trained to escape to a visible stable platform (NW quadrant). TP: visible stable platform replaced by a visible unstable platform (NW quadrant) and a new hidden platform was added (SE quadrant); animals were trained to inhibit escape to the visible unstable stable platform (NW quadrant) and locate the hidden stable platform.

The test phase started the following day. During this phase, the visually cued rigid platform was converted to a visually cued floating platform, which was otherwise identical in appearance. A new hidden rigid platform was added to the opposite quadrant approximately 0.5cm below the surface of the water. The room contained numerous distal visual cues including shelves, cabinets and posters distributed throughout the room. The test phase consisted of two consecutive trials for eight consecutive days. Each trial terminated at 45s or upon the animal locating the new hidden platform. Animals that did not find the new hidden (stable) platform were guided to it and allowed to rest atop the platform for 15s before being removed to a warm dry towel for 30s between trials. A schematic of the apparatus used in the acquisition and test phase is presented in Figure 1.

### Blood Ethanol Concentration (BEC)

Parallel groups of animals (acute study: n=8; chronic study: n=8) were used to assess initial BEC. For the acute study, animals were dosed on PND34 (corresponding to the first day of the test phase) with 1g/kg ethanol (i.p., 16% v/v in normal saline). For the chronic study, animals were dosed (i.g.) on PND30 (corresponding to the first day of chronic exposure) with 5g/kg ethanol (35% v/v in normal saline). In both groups, blood (~300μL) was drawn from the lateral saphenous vein at 30 minutes post (acute study) or 60 minutes post (chronic study) administration. Serum was collected from centrifuged samples and stored at -80°C. Ethanol concentration was analyzed in triplicate using an Analox GL5 alcohol analyzer (Analox Instruments, Lunenburg, MA). 

### Statistical Analyses

Descriptions of each behavioral variable are presented in Table 1. Latency was used instead of distance as the primary dependent measure because distance did not adequately reflect perseverative behavior. That is, some animals spent considerable time trying to mount the unstable floating platform during the test phase, but in doing so, amassed relatively little total swim distance. Non-spatial learning was assessed using the latency to the visible platform (acquisition phase).

**Table 1:** 

Variable	Description and Operational Definition
Latency (acquisition phase)	Time elapsed from release to escape on the platform
Thigmotaxis	Total time animal spent in the concentric zone at the outer edge of the tank
Speed	Distance / time
Perseveration time	Time animals spent attempting to mount the hidden platform
Head Entries	Number of times an animal's head entered the concentric zone around the floating platform
Extinction Failure	Proportion of animals that return to the floating platform on a given day
Inefficiency	Cumulative time or distance traveled to the floating platform on those days it was visited first
Latency (test phase)	Time elapsed from release to escape on the hidden platform, minus perseveration time

Thigmotaxis and speed were assessed during the test phase as control variables. They were assessed to provide insight into non-cognitive processes such as anxiety and gross locomotor function, respectively. Disinhibition was assessed during the test phase using the time animals spent attempting to mount the floating platform, the number of entries into the floating platform zone, and the proportion of animals in each group that swam to the floating platform on each day. The duration and number of attempts to mount the floating platform were taken as direct measures of perseverative behavior.

We also assessed the efficiency with which animals reached the floating and hidden platforms using latency, distance traveled, and swim speed to the respective platform. During experiment 1, only one ethanol-treated animal swam directly to the hidden platform on both trials of a given day. Therefore, there was insufficient data in experiment 1 to analyses the effect of acute ethanol on behavioral efficiency in approaching the hidden platform and so it was analyzed in experiment 2 only. 

Spatial learning was assessed during the test phase using the latency to the hidden platform. The latency to find the new hidden platform was adjusted to eliminate the time spent trying to mount the floating platform. This correction was made by subtraction of total perseveration time from total latency in order to distinguish disinhibition from spatial learning. Animals that did not reach the new hidden platform were assigned a maximum trial latency of 45s. 

Data from the acquisition and test phases were analyzed independently using a repeated measures analysis of variance. Independent variables included Session (acquisition phase) or Day (test phase) as the repeated measure and drug group (ethanol vs. saline) as the between group independent variables. All analyses were performed with SPSS v18 (IBM, Chicago). The sphericity assumption was tested using Mauchly’s W for all repeated measures. Degrees of freedom were adjusted using Greenhouse-Geisser correction where Mauchly’s W was significant (p<0.05) and Lower-bound where Mauchly’s W was undefined. Ordinal interactions were followed by tests of simple main effects. Cochran’s Q, a non-parametric test for repeated measures, was used to assess changes in the proportion of animals that fail to extinguish the pre-potent behavior across days. This method was chosen due to the absence of any widely accepted means of testing changes in proportions in a mixed model design. In an attempt to be conservative, we first collapsed the ethanol and saline groups together and tested the hypothesis that there was a decline in the proportion of animals failing to extinguish the pre-potent response. If this proved significant, we then separated the ethanol and saline groups and re-ran Cochran’s test to determine if the decline was present in both groups. SPSS truncates observed probabilities at three decimal places. SPSS generated probabilities of 0.000 are reported as p<0.001. Alpha was set at p<0.05 for all analyses. 

## Results

Acute doses of ethanol during adolescence promoted disinhibition as well as behavioral inefficiency. Interestingly, although CIE administered during adolescence had no lasting effect on disinhibition, it did promote behavioral inefficiency under circumstances in which animals swam to the floating platform instead of the hidden platform. When animals bypassed the floating platform and swam directly to the hidden platform there was no evidence of an effect of CIE. 

### Experiment 1

**Figure 2:**
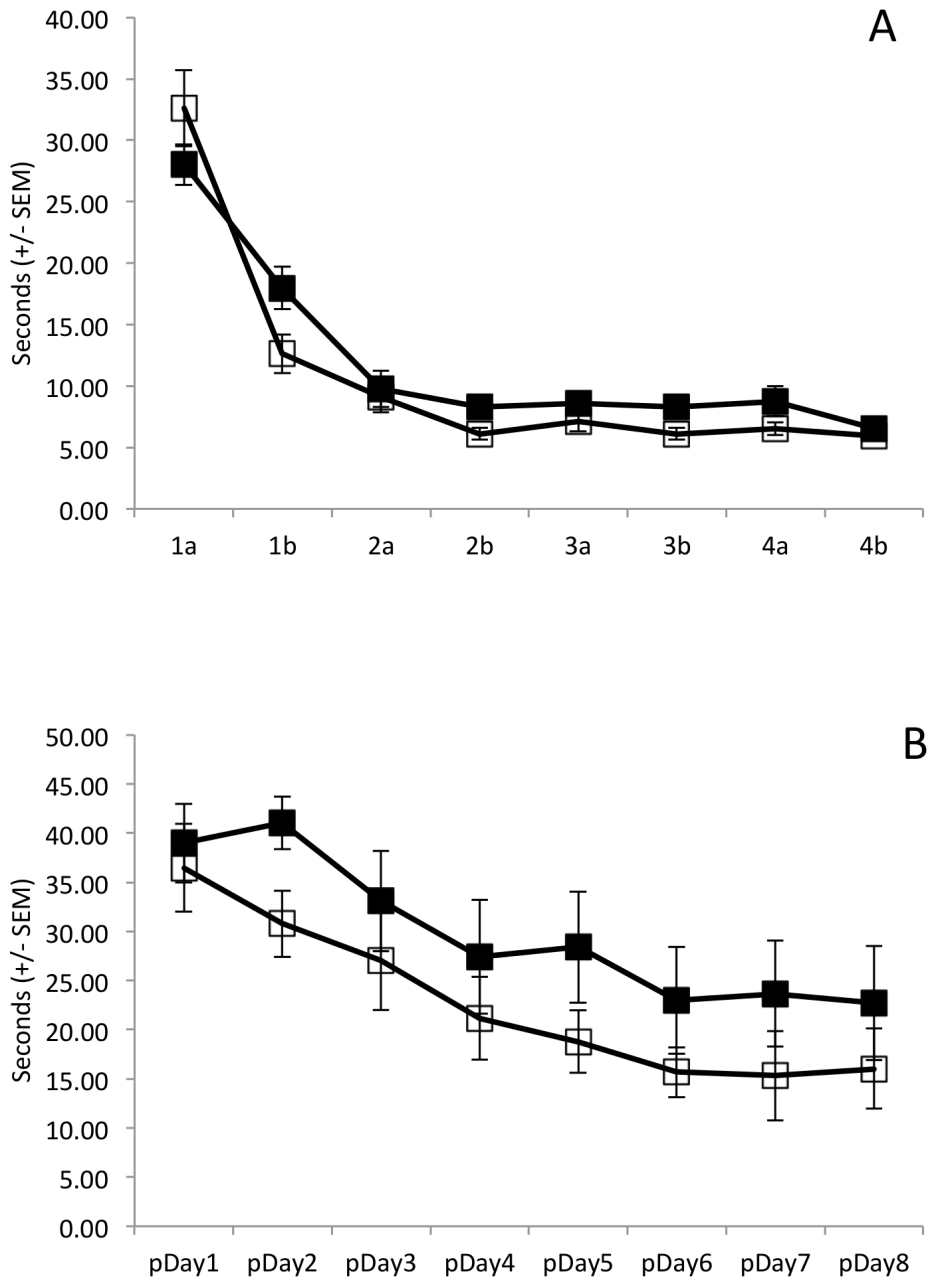
Escape latency (seconds +/- SEM), experiment 1. Acute ethanol (1.0g/kg; solid squares) or saline (open squares) was administered 30 minutes prior to each training session during the test phase. A) Non-spatial learning was significant across daily sessions (1a, 1b, 2a, 2b…, p<0.001); no significant difference on baseline non-spatial learning between groups that would receive ethanol during test phase (p=0.07). B) Controlling for perseveration, spatial learning of the new hidden platform declined significantly across perseveration days (pDay1, pDay2…; p<0.001); learning across days did not differ between groups (p=0.95); groups did not differ in cumulative performance (p=0.17).

During the acquisition phase (Figure 2a), all animals learned the location of the visible platform (main effect of session: F(7,98)=79.73, p<0.001). Although animals were not treated with ethanol or saline during the acquisition phase, we assessed the presence of baseline group differences in initial learning. Collapsing across acquisition session, there was no difference between those animals that would later receive ethanol and those that would receive saline in the reversal phase (main effect of Group: F(1,14)=4.01, p=0.07). Moreover, rate of acquisition did not differ by group (Group x Day interaction: F(7,98)=2.34, p=0.149). 

Based on results from a parallel group of animals, we estimated that animals receiving 1g/kg ethanol (i.p.) during the test phase achieved BECs of approximately 0.09g/dL (SEM, 0.026) at the time the behavioral testing was initiated. Acute ethanol produced only minor non-specific behavioral effects during this phase. Animals were more thigmotaxic than controls on Day 1 of the test phase, but not Days 2 - 8. The duration of thigmotaxic behavior declined in a group dependent manner across days (Day x Group interaction: F(7,98)=3.0, p=0.03). Simple main effects of Day reveal that thigmotaxis declined across days among ethanol treated animals (F(7,49)=3.17, p=0.008), but not among saline treated animals (F(7,49)=0.97, p=0.46). Simple main effects of Group reveal that saline and ethanol treated animals differed only on day 1 (F(1,14)=8.1, p=0.01). In addition, animals receiving acute ethanol swam slower than controls (main effect of Group: F(1,14)=4.73, p=0.05). There was no change in swim speed across days (main effect of Day: F(7,98)=1.91, p=0.13) and no group dependent change in swim speed (Group x Day interaction: F(7,98)=1.23, p=0.31). 

**Figure 3:**
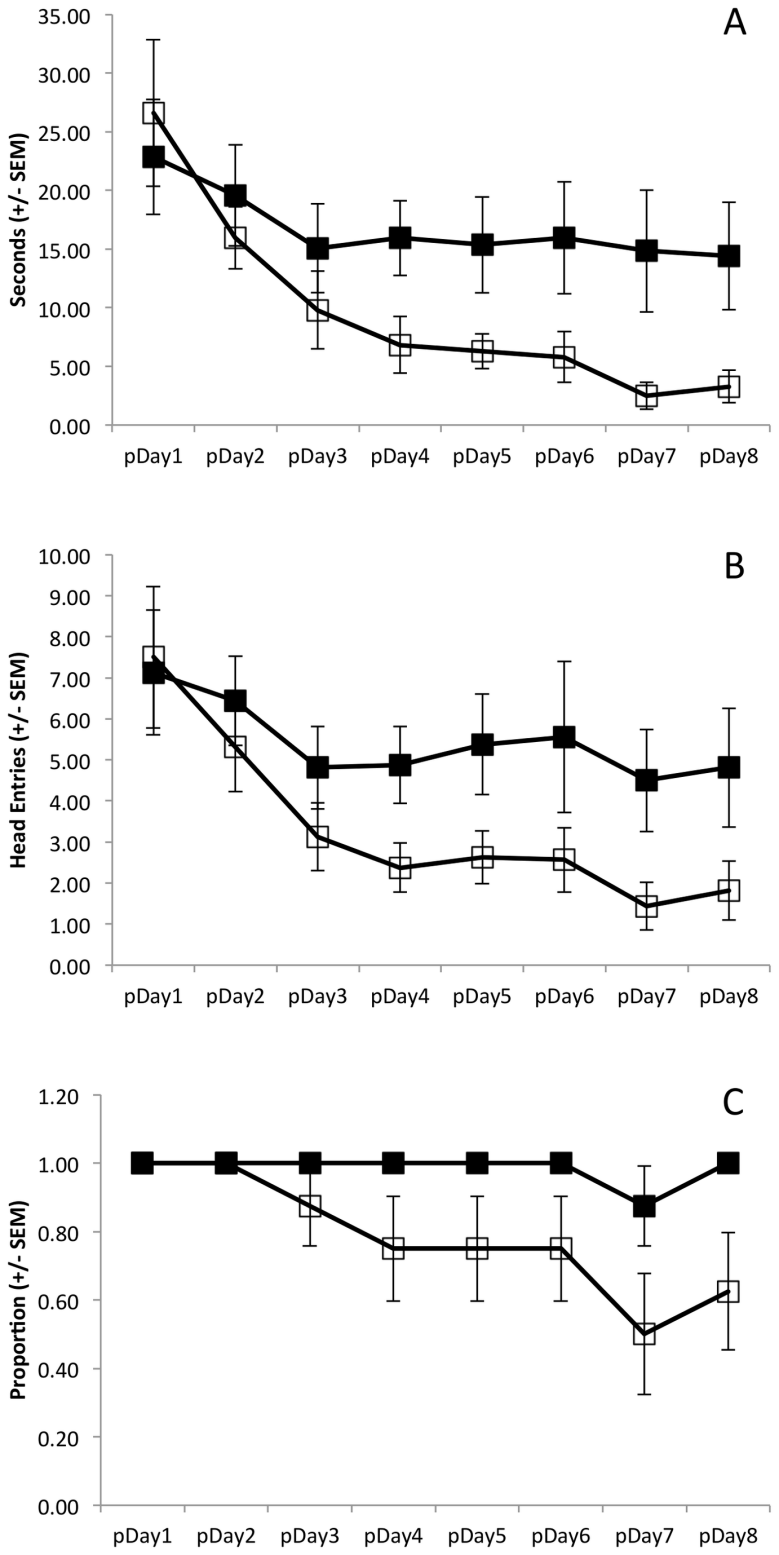
Measures of disinhibition during the test phase by day (pDay1, pDay2…; acute ethanol: solid squares; saline: open squares). Ethanol treated animals spent more time (A; p=0.047) and made more attempts (B; p=0.03) trying to mount the floating platform than saline treated animals. C) The proportion of animals that failed to extinguish the pre-potent response declined in the saline group (p=0.04) but not in the ethanol group (p=0.54).

During the test phase, animals treated with ethanol spent more time attempting to mount the unstable platform (Figure 3a) and continued to make more head entries into the floating platform zone (Figure 3b) than did controls. Collapsing across days, ethanol treated animals spent more time trying to mount the floating platform than saline treated animals (main effect of Group: F(1,14)=4.76, p=0.047). The time spent attempting to mount the unstable platform (collapsed across groups) decreased across days (main effect of Day: F(7,98)=5.86, p=0.003). However, this decrease in perseveration time did not differ as a function of group (Group x Day interaction: F(7,98)=1.36, p=0.27). Moreover, there was a significant decline in the number of head entries into the floating platform zone across days (main effect of Day: F(7,98)=4.69, p=0.005; Figure 3b). While this decline did not differ as a function of group (Group x Day interaction: F(7,98)=0.81, p=0.5), ethanol treated animals clearly made more entries into the floating platform zone than did the saline treated animals on Days 4 through 8 (main effect of Group: F(1,14)=6.3, p=0.03). No group effect was present during the initial three days of the spatial acquisition phase (main effect of Group: F(1,14)=0.47, p=0.51). 

**Figure 4:**
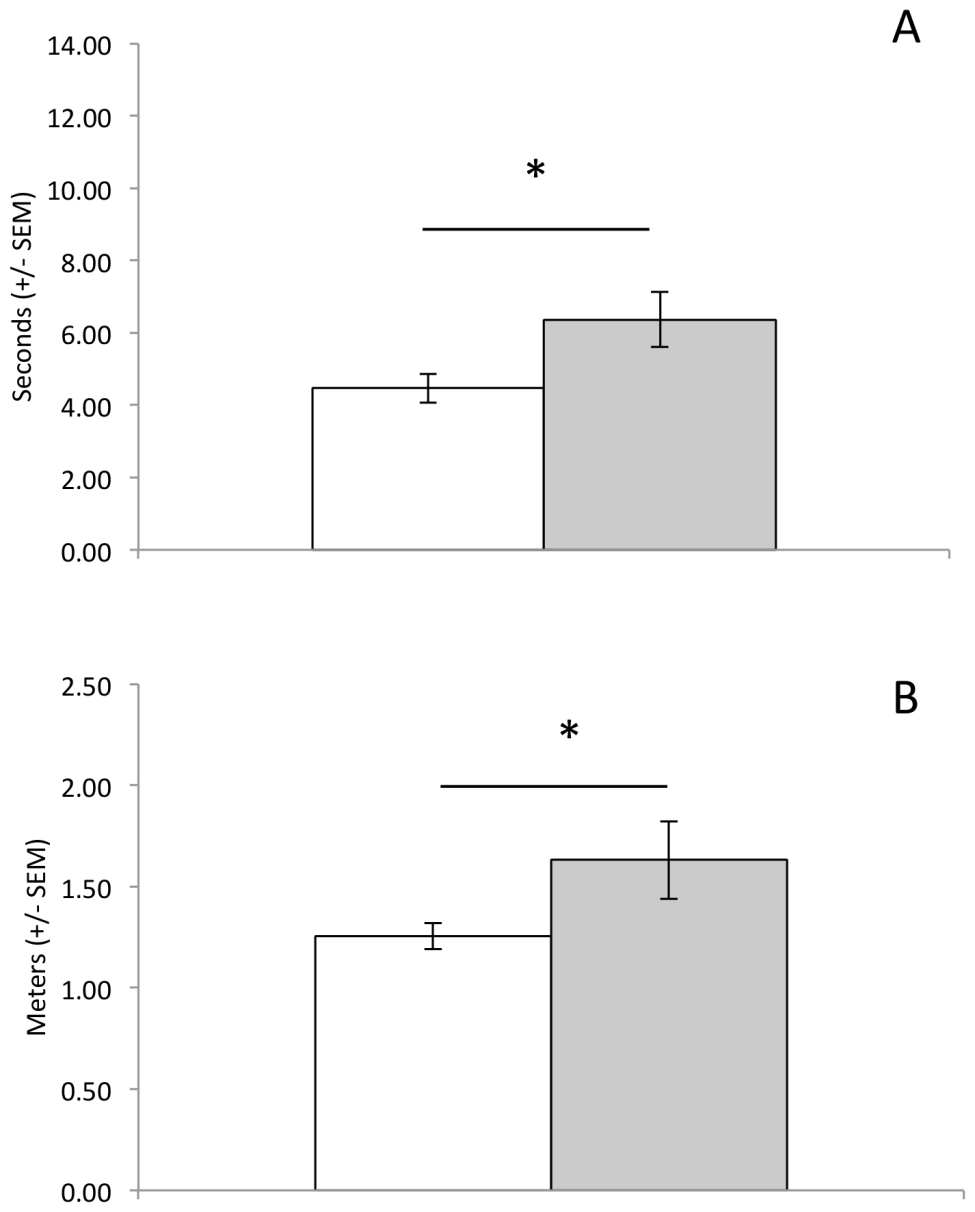
Measures of inefficiency during the test phase (acute ethanol: gray bars; saline: open bars). Ethanol treated animals swam longer (A) and farther (B) before reaching the floating platform on those days on which they swam to the floating platform. * p<0.05.

In addition, the proportion of animals that failed to extinguish the pre-potent behavioral response to swim to the visible platform declined in the saline treated group, but not the ethanol treated group (Figure 3c). Collapsing groups together, there was a significant decline in the proportion of animals swimming to the floating platform across days (main effect of Day: Q(7)=19.05, p=0.008). However, this decline appears to have been driven by the decline in the proportion of saline treated animals swimming to the floating platform (main effect of Day within saline Group: Q(7)=14.54, p=0.04). There was no significant decline in the proportion of ethanol treated animals that went to the floating platform (main effect of Day within ethanol Group: Q(7)=6.0, p=0.54). On the days on which an animal swam to the floating platform (Figure 4a - 4b), ethanol treated animals took more time (t(14)=2.27, p=0.02) and swam farther (t(14)=1.85, p=0.05) before reaching the floating platform than did saline treated animals. 

Although the latency to find the new hidden platform was consistently longer for ethanol treated animals than for saline treated animals (Figure 2b), the group effect did not reach statistical significance (main effect of Group: F(1,14)=2.07, p=0.17). Animals in both groups learned to find the hidden platform (main effect of Day: F(7,98)=11.35, p<0.001) and there was no group dependent difference in this learning (Group x Day interaction: F(7,98)=0.3, p=0.95). 

### Experiment 2

Based on results from a parallel group of animals, we estimate that animals receiving 5g/kg ethanol (i.g.) during the ethanol exposure phase achieved BECs of approximately 0.23g/dL (SEM, 0.055), measured 60 minutes following administration. These blood ethanol concentrations are in keeping with BECs achieved by adolescent humans during binge drinking episodes and serves as part of the defining criteria of binge drinking [37].

**Figure 5:**
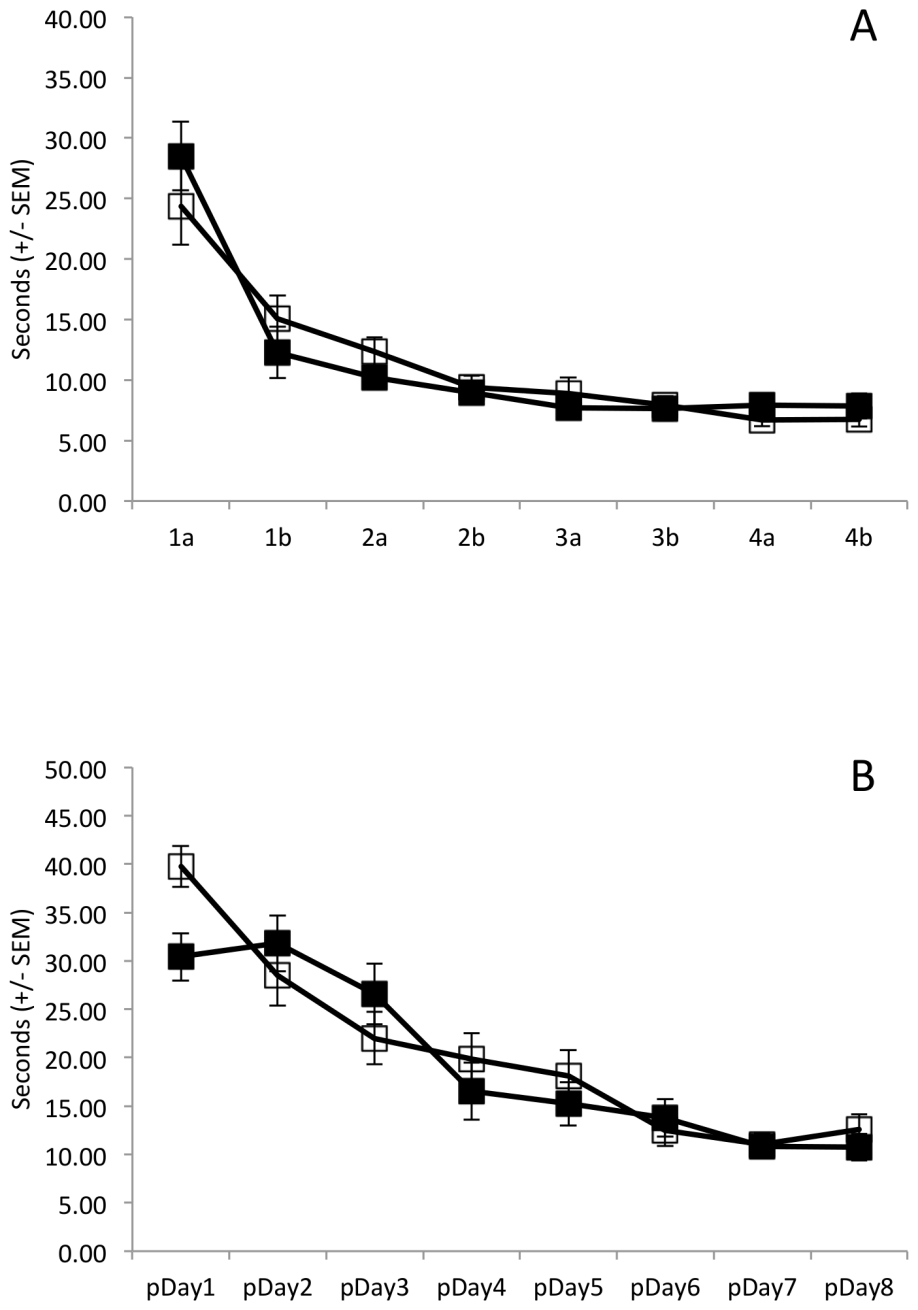
Escape latency (seconds +/- SEM), experiment 2. Chronic adolescent ethanol exposure (5.0g/kg; solid squares) or saline (open squares) was administered between PND30-PND45 followed by washout (PND46-PND71). A) Non-spatial learning was significant across daily sessions (1a, 1b, 2a, 2b…p<0.001); no significant difference on baseline non-spatial learning between groups (p=0.97). B) Controlling for perseveration, spatial learning of the new hidden platform declined significantly across perseveration days (pDay1, pDay2… p<0.001); learning across days did not differ between groups (p=0.07); groups did not differ in cumulative performance (p=0.57).

During the acquisition phase (Figure 5a), the latency required to reach the visible platform decreased among all animals (main effect of Day: F(7,154)=44.66, p<0.001). Moreover, the decline in latency did not differ as a function of group (Group x Day interaction: F(7,154)=1.3, p=0.28) and there was no cumulative difference in performance between the CIE pre-treated and saline pre-treated animals (main effect of Group: F(1,22)=0.001, p=0.97). 

Analyses of control variables (thigmotaxis and speed) from the test phase reveal a significant decline in thigmotaxis across days (main effect of Day: F(7,154)=9.4, p<0.001) with no effect of CIE pre-treatment (main effect of Group: F(1,22)=0.45, p=0.51), and no group dependent decline in thigmotaxis (Group x Day interaction: F(7,154)=0.36, p=0.78). Swim speed did not change across days (main effect of Day: F(7,154)=1.56, p=0.15) or between groups (main effect of Group: F(1,22)=0.38, p=0.38). The interaction between day and group was also non-significant (F(7,154)=1.34, p=0.24). 

**Figure 6:**
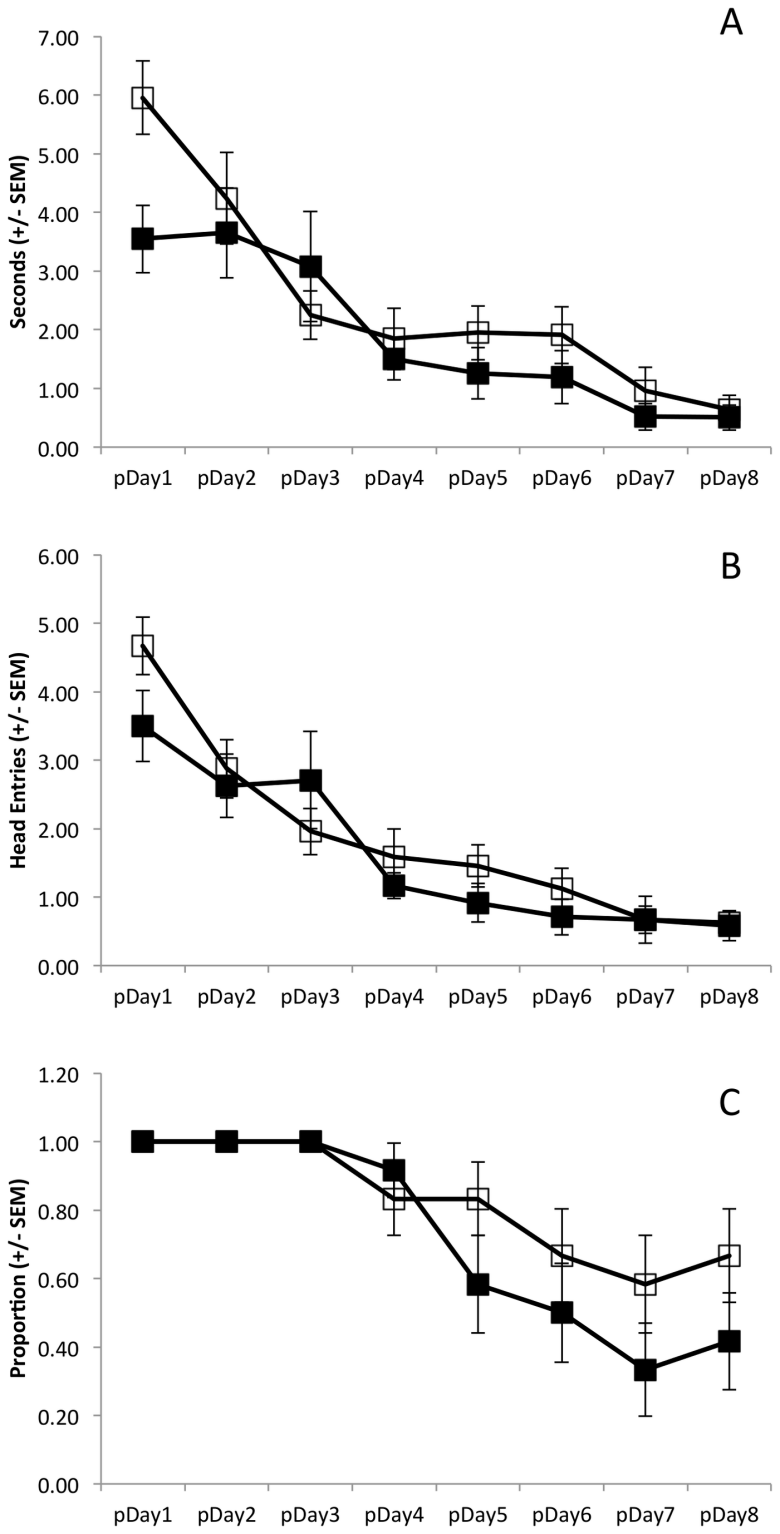
Measures of disinhibition during the test phase by day (pDay1, pDay2…; chronic ethanol: solid squares; saline: open squares). Ethanol and saline treated animals did not differ in the time (A; p=0.3) or number of attempts (B; p=0.36) made trying to mount the floating platform. The time (p<0.001) and number (p<0.001) of entries declined across days in both groups. C) The proportion of animals that continued to mount the floating platform declined in both the saline (p<0.001) and CIE (p<0.001) pre-treated groups.

Data from the test phase reveal a decline across days in the time animals spent attempting to mount the floating platform (main effect of Day: F(7,154)=19.94, p<0.001; Figure 6a). This decline was not Group dependent (Group x Day interaction: F(7,154)=0.18, p=0.954) and there was no cumulative difference between the CIE and saline pre-treated groups (main effect of Group: F(1,22)=1.14, p=0.3). Results from data concerning the number of head entries into the floating platform zone were identical (Figure 6b). There was a decline in the number of head entries across days (main effect of Day: F(7,154)=26.04, p<0.001), but this effect was not group dependent (Group x Day interaction: F(7,154)=1.3, p=0.28) and there was no cumulative difference between the CIE and saline pre-treated groups (main effect of Group: F(p=0.36). Data concerning the proportion of animals from each group that swam to the floating platform on each day (Figure 6c) reveal an overall decline in the number of animals that visit the floating platform (main effect of Day: Q(7)=56.55, p<0.001). Moreover, this decline was significant in both the saline (main effect of Day within saline Group: Q(7)=19.96, p=0.006) and the CIE pre-treated (main effect of Day within ethanol Group: Q(7)=38.19, p<0.001) animals. 

**Figure 7:**
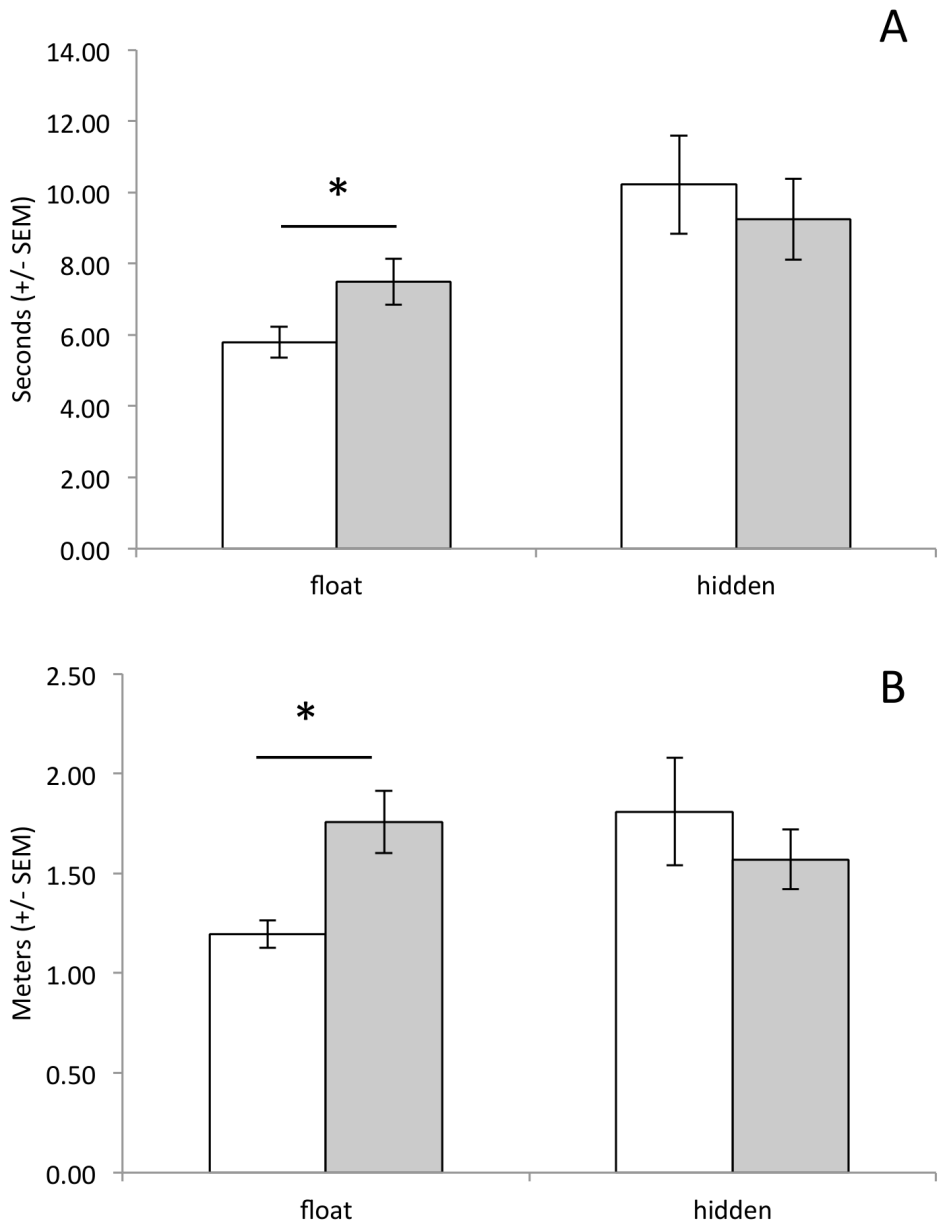
Measures of inefficiency during the test phase (chronic ethanol: gray bars; saline: open bars) as animals swim to either the floating (left panel) or hidden (right panel) platforms. Animals treated with chronic ethanol during adolescence swam longer (A) and farther (B) before reaching the floating platform on those days on which they swam to the floating platform. There was no difference between groups in behavioral efficiency when they swam directly to the hidden platform. * p<0.05.

Although CIE pretreated animals failed to show signs of behavioral disinhibition, they were less efficient in their behavior, as we observed in experiment 1 after acute ethanol. That is, CIE pre-treated animals were significantly less efficient than saline pre-treated animals when it came to locating the floating platform (Figure 7). CIE animals swam longer (t(22)=2.16, p=0.02) and further (t(22)=3.3, p=0.002) than saline animals before reaching the floating platform, on those days on which they swam to the floating platform first. However, there was no such inefficiency when animals swam directly to the hidden platform (latency: t(14)=0.55, p=0.3; distance: t(14)=0.83, p=0.21). 

After correcting for the time spent trying to mount the floating platform, there was clear evidence that all animals learned the location of the new hidden platform (Figure 5b) during the test phase (main effect of Day: F(7,154)=36.05, p<0.001). However, this learning was not influenced by pretreatment with CIE (Group x Day interaction: F(7,154)=2.15, p=0.07) and there was no cumulative effect of CIE pretreatment across days (main effect of Group: F(1,22)=0.34, p=0.57). 

## Discussion

Perseverative behavior is generally recognized as a failure of response inhibition, the latter being a component of a larger set of skills referred to as executive functions. Such functions are subserved by the frontal lobes and their interconnections with other cortical and subcortical regions. Although the detrimental effects of ethanol on executive functions are well established, and adolescence is the developmental period during which executive functions emerge [13,38], little is known about the effects of adolescent alcohol exposure on these critical functions. Therefore, the purpose of this study was to determine if ethanol administered during adolescence promotes perseverative behavior either acutely, or later in adulthood following chronic ethanol exposure during adolescence. 

We found that an acute dose of 1.0g/kg ethanol promoted perseveration in adolescent animals, consistent with previous studies showing that 1.5 and 2.0 g/kg (but not 0.75 or 1.0g/kg) ethanol promote perseveration in adult animals in a radial arm maze task [5,6]. Interestingly, the discrepancy between the effect of 1.0g/kg ethanol in adolescent animals (present study) and adult animals [5] may suggest greater sensitivity among adolescents to the promotion of perseveration by acute ethanol. This would be consistent with much of the existing work on the neurodevelopmental effects of ethanol in adolescence indicating that ethanol has more marked effects on some behavioral measures in adolescent rats, compared to adults (see 39). However, further studies making direct comparisons between adolescents and adults will be required before such a conclusion can be justified.

We also found that animals under the influence of ethanol approach the floating platform less efficiently than saline treated animals. That is, ethanol treated animals swim longer and farther before reaching the floating platform than saline treated animals. This could reflect general inefficiency of cognitive or behavioral function, or it could be specific to situations in which there is some behavioral conflict. In this task the conflict could have been between returning to the floating platform and inhibiting that pre-potent response in favor of seeking a new means of escape. It is significant that acute ethanol produced this search inefficiency in the context of a challenging task involving both behavioral inhibition and cognitive flexibility. Deficits in behavioral efficiency are often associated with the effects of chronic ethanol exposure (see discussion below) and suggest that acute ethanol influences similar processes in adolescent animals. 

Although acute ethanol increased escape latency relative to saline consistently throughout the spatial learning phase, it is noteworthy that this ethanol effect did not reach statistical significance. We have previously shown that 1.0g/kg ethanol impaired spatial memory acquisition in adolescent animals [22]. However, this discrepancy is likely explained by task related variables. Most important among these variables, our previous study assessed the effects of ethanol on initial acquisition of the spatial learning task whereas the present study allowed initial acquisition in a cued learning condition prior to the assessment of spatial learning and perseveration. It is now well established that prior swimming experience affects the water maze learning process by way of basic sensory-motor function [40-42]. In addition, the spatial learning task in our earlier study may have been more demanding than that in our present study. Search area (i.e., diameter of the tank) is a critical determinant of the difficulty of the water maze task with larger search areas placing greater demands on spatial learning processes [43]. The tank used in the present study was slightly smaller than that used previously (1.5 vs. 1.7m) and the effective search distance in the test phase of the present study (where we measured spatial acquisition) was constrained by the narrower distance between the floating platform and the new hidden platform (~1 meter). As a result, the spatial acquisition component of the present study may not have been sufficiently sensitive to detect the effect of 1.0g/kg ethanol in these adolescent animals. 

Nonetheless, we would be remiss to ignore other recent studies that suggest ethanol has no differential effect on spatial learning in adolescent and adult animals [29-32]). While the results of these studies seem inconsistent with our earlier report [22], their comparability with our earlier work is limited. For example, work from Matthews’ group [29] has focused on the effect of ethanol on spatial memory recall rather than spatial learning or acquisition. Similarly, Hefner and Holmes ([32]) used both a different species and a different behavioral task. Given the evidence that mice and rats manifest very different pharmacokinetic profiles in response to ethanol [44], the single 2.0g/kg dose administered would not have been sufficient to test the hypothesis that the effect of ethanol on fear conditioning is age dependent. There is also good evidence that spatial learning in the water maze and fear conditioning rely on distinct neural substrates, which further limits the comparability of that study with our previous work [22]. Finally, the sandbox task [31] was not methodologically or analytically well described in the conference proceedings in which it was published and may be open to alternate interpretations (e.g., use of olfactory cues cannot be ruled out). In addition, the one-meter diameter of the sand box arena makes this task even less sensitive as a measure of spatial learning than the 1.5m tank used in the present study. 

Results from Experiment two demonstrated that CIE during adolescence produced no significant effect on learning or perseveration when animals were assessed as adults (PND73). This is consistent with our recent study showing that CIE during adolescence or adulthood had no effect on subsequent baseline spatial learning in the radial arm maze [45]. However, CIE during adolescence did promote behavioral inefficiency in the context of competing goals. That is, when animals swam first to the non-reinforced floating platform, CIE pre-treated animals swam longer and farther than saline pre-treated animals before reaching it. On days in which animals swam first to the hidden (reinforced) platform, there was no difference between pre-treatment groups in the latency or distance swam before reaching the platform. This may reflect a deficit in adaptive decision-making, which has been associated with CIE. For example, in late adolescent/young adult humans, maladaptive decision making on the Iowa Gambling Task has been shown to be related to heavy, but not moderate binge-pattern drinking [46]. Interestingly, the association was independent of measures of impulsivity or the age of onset of drinking. 

However, the deficit observed in the present study does not appear to involve adaptive decision making directly (i.e., which platform to swim to). There was no difference in the proportion of animals in each group that swam to the floating platform across days. Rather, the deficit appears limited to those trials on those days in which animals swam to the floating (non-reinforced) platform. 

We interpreted this ethanol-induced deficit as a form of behavioral inefficiency because it is the most face-valid explanation for the observed behavior. Efficiency generally refers to the time or energy needed to accomplish a particular task. Highly efficient behaviors involve the completion of tasks in little time or having consumed little energy. In the current model, the task involved swimming to the floating platform. CIE pre-treated animals spent more time and traveled further than saline pretreated animals to reach the same goal location. Hence, the behavior of CIE pre-treated animals was less efficient than that of saline pre-treated animals.

Ethanol-related impairments in cognitive efficiency have been previously reported in the human literature (e.g., [47-52]). Within the human ethanol research literature, efficiency has been defined as “the capacity to utilize accurate or relevant information while ignoring or disregarding inaccurate or irrelevant information” [50] and deficits thereof are demonstrated by “an inability to ignore irrelevancy” [47]. Such definitions appear to capture the essence of the behavior observed in our current study. Importantly, we observed the deficit in efficiency only in circumstances where the animal swam to the non-reinforced platform. No such inefficiency was observed when animals swam directly to the hidden platform. The precise nature and corresponding neurobiology for such a deficit remains unclear and is in need of further study. Nonetheless, this is an important observation concerning the long-term consequence of adolescent CIE exposure because it may lead to a more fine-grain understanding of the nuances of behavioral alterations after CIE. It is also significant because there have been relatively few learning-related changes in behavior after CIE independent of additional challenges (see [45]). 

In addition to the empirical findings in this study, it is notable that the behavioral methodology used was modified from the standard usage of the water maze. Similar to other tasks designed to elicit perseverative behavior (e.g., operant lever press [53]; spontaneous alternation [54]; and, 5-choice serial reaction time tasks [55]), animals in this task are trained to develop a pre-potent response (finding the visible platform) and then the task demands change such that the previous behavior must be inhibited. It is significant that adolescent control animals (Figure 3a) spent nearly five times longer trying to mount the floating platform than did the adult controls (Figure 6a). This is consistent with the relative immaturity of frontal executive systems in adolescent relative to adult humans [10,11]. This is also consistent with findings that adolescent rats are more impulsive than adult rats in the delayed discounting task [56].

The floating platform methodology used here is not entirely novel. Indeed, Morris [57] used a floating platform early in his development of this now ubiquitous behavioral assay. However, Morris and others used the floating platform as a means of testing discrimination learning. The present model differs from those earlier uses of the floating platform in that we first trained animals on the location of the visible platform and then converted it to a floating platform. While this task is akin to traditional water maze reversal tasks, the addition of the floating platform during the reversal phase markedly increases saliency of the former platform location and therefore increases the probability of perseverative behavior. Thus, it models the kind of perseveration to a pre-potent response that is often observed clinically. Moreover, it is modeled in a task that can be easily adapted from the traditional water maze task used by many laboratories and requires far less time (~13 days) than tasks such as 5-CSRT (> 40 days; [58]). 

These data indicate that adolescent animals are significantly more prone to repetitive, non-instrumental behavior following an acute low dose of ethanol. Assuming such effects scale up to adolescent humans, an ethanol induced absence of behavioral flexibility would put the individual at increased risk in complex, cognitively demanding situations. This effect, in conjunction with developmentally normal risk taking behavior (see [36] for review), may well contribute to the many adverse behavioral sequelae of alcohol intoxication in adolescents and young adults. Moreover, a high propensity toward perseverative behavior in adolescents under the influence of ethanol could also lead to greater ethanol consumption; increased probability of the acute risks of high intoxication; as well as an increased liability to ethanol addiction. 
